# Pulmonary sarcoidosis-like reactions induced by sintilimab in esophageal cancer: A case report

**DOI:** 10.1097/MD.0000000000034432

**Published:** 2023-08-04

**Authors:** Haoqian Li, Fengchun Mu, Bing Zou, Linlin Wang

**Affiliations:** a Department of Oncology, Shandong First Medical University and Shandong Academy of Medical Sciences, Jinan, Shandong, People’s Republic of China; b Department of Radiation Oncology, Shandong Cancer Hospital and Institute, Shandong First Medical University and Shandong Academy of Medical Sciences, Jinan, Shandong, People’s Republic of China.

**Keywords:** adverse drug reactions, case report, Esophageal cancer, monoclonal antibodies

## Abstract

Esophageal cancer is one of the deadliest cancers in the world, with high incidence and mortality rates ranking among the top ten in China. The efficacy of conventional treatments is limited and often accompanied by severe adverse reactions, which results in unsatisfactory outcomes. The mechanism of immune checkpoint inhibitors (ICIs) is to activate cytotoxic T cells to kill tumor cells expressing tumor antigens. The application of ICIs has profoundly changed the mode of cancer treatment. However, the use of ICIs also induces a series of adverse reactions similar to autoimmune reactions, called immune-related adverse events (irAEs). Some ICIs can cause manifestations similar to those in the development of sarcoidosis, which are called sarcoidosis-like reactions or granulomatosis.

**Patient concerns:**

We report a 50-year-old Chinese male patient.

**Diagnoses:**

The patient had been diagnosed with advanced esophageal squamous cell carcinoma , and was confirmed to have pulmonary sarcoidosis-like reactions associated with sintilimab, a human programmed cell death protein 1 (PD-1) inhibitor.

**Interventions:**

The patient was administered corticosteroid treatment.

**Outcomes:**

After receiving steroid treatment, the patient’s systemic and pulmonary symptoms improved rapidly. To our knowledge, this is the first report of pulmonary sarcoidosis-like reaction in a patient with esophageal squamous cell carcinoma. The patient then continued to receive 1 year of follow-up antitumor treatment after the appearance of lung pulmonary sarcoidosis-like reactions. The prognosis was good and the patient’s condition is currently stable.

**Lessons:**

The diagnosis of ICI-induced sarcoidosis often requires comprehensive evaluation through clinical, pathological, and radiological assessment. A subset of patients with sarcoidosis-like reactions may not require treatment unless there is organ dysfunction or severe clinical symptoms, and these reactions generally respond well to treatment. The occurrence of sarcoidosis-like reactions after immunotherapy is positively correlated with the long-term prognosis of cancer patients. However, this hypothesis requires larger prospective studies for validation.

## 1. Introduction

Esophageal cancer is one of the most lethal cancers in the world, and the morbidity and mortality rank among the top 10 in China. At present, the means of treatment are surgical resection, radiotherapy and chemotherapy. However, due to the limited efficacy of routine treatment and serious adverse reactions, the outcomes are still unsatisfactory. Immune checkpoint molecules are promising anticancer targets in recent years. Among them, the immune checkpoint antibodies which inhibit programmed cell death protein 1 (PD-1)/PD-Ligand1 pathway have shown activity against a variety of malignant tumors, and has been widely used in the treatment of unresectable or metastatic solid tumors.^[1]^ PD-1 inhibitor Sintilimab is a highly selective recombinant humanized anti-PD-1 antibody, which can specifically block the interaction between PD-1 and its ligands, and has stronger affinity for human PD-1 than other PD-1 inhibitor such as Nivolumab and Pembrolizumab.^[2–4]^ Therefore it has been approved as a standard therapeutic drug for the treatment of programmed death ligand 1 positive or advanced esophageal squamous cell carcinoma. Unfortunately, immunotherapy can also cause some unexpected side effects called Immune-related adverse events (irAEs), which refers to a series of immune checkpoint inhibitors (ICI)-induced effects similar to autoimmune reactions. It has been proved that some ICIs can cause symptoms similar to those in the development of sarcoidosis, which are called “sarcoidosis-like reactions”. The etiology of sarcoidosis-like reactions is still unknown. Biopsy shows non-caseous granuloma, and patients may suffer from severe systemic manifestations.^[5,6]^

## 2. Case report

**Figure 1.**
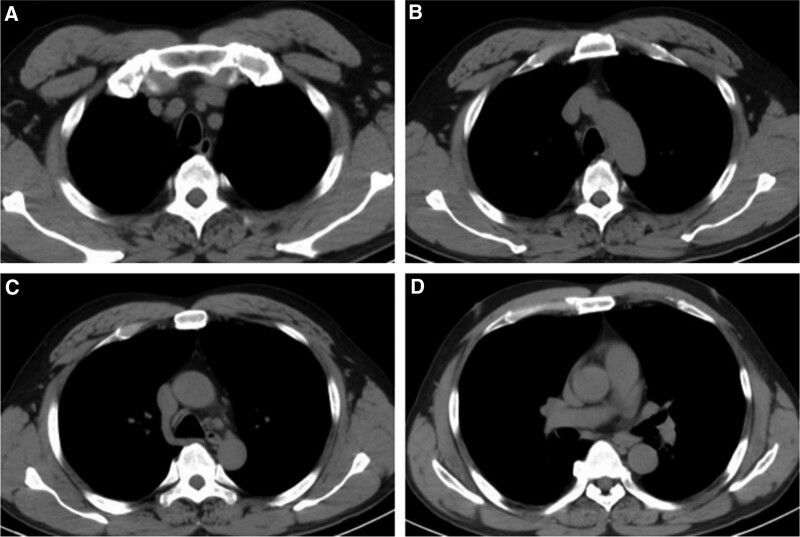
Computed tomography before the administration of immune treatment showing slight enlargement of mediastinal and bilateral hilar lymph nodes.

**Figure 2.**
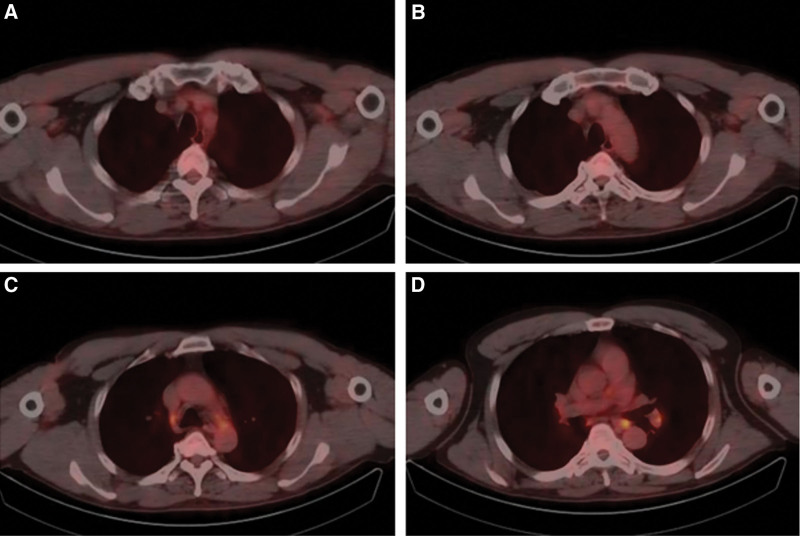
18F-FDG-PET imaging in September 2021 demonstrating slight hypermetabolic mediastinal and bilateral hilar lymph nodes. FDG = fluorodeoxyglucose, PET = positron emission tomography.

A 50-year-old man, with no previous medical history, was diagnosed in September 23, 2021 with lower thoracic esophageal carcinoma (pathological squamous cell carcinoma, imaging stage cT3N2M1, stage IVB) with left supraclavicular, mediastinal, abdominal and retroperitoneal lymph node metastasis and fluorodeoxyglucose hypermetabolism (Figs. 1, 2).

**Figure 3.**
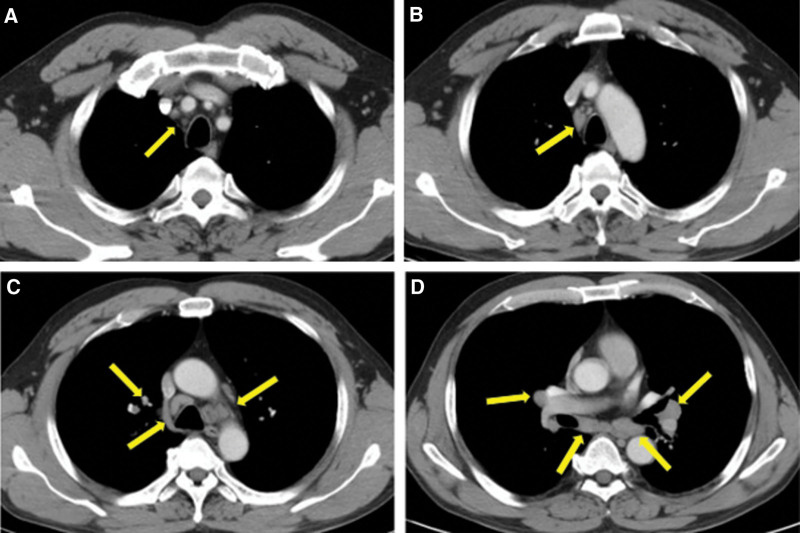
Computed tomography scan after two cycles of sintilimab treatment of the case report patient showing multiple enlargement of mediastinal and bilateral hilar lymph nodes (yellow arrows).

Multidisciplinary consultation suggests systemic treatment first and palliative radiotherapy at the right time. The patients acquired a treatment with sintilimab combined with chemotherapy and radiotherapy. Sintilimab 200 mg was administrated at 3-week intervals. After 2 cycles of treatment, computed tomography (CT) scan showed multiple enlarged lymph nodes in bilateral hilum, mediastinum and supraclavicular areas (Fig. 3).

**Figure 4.**
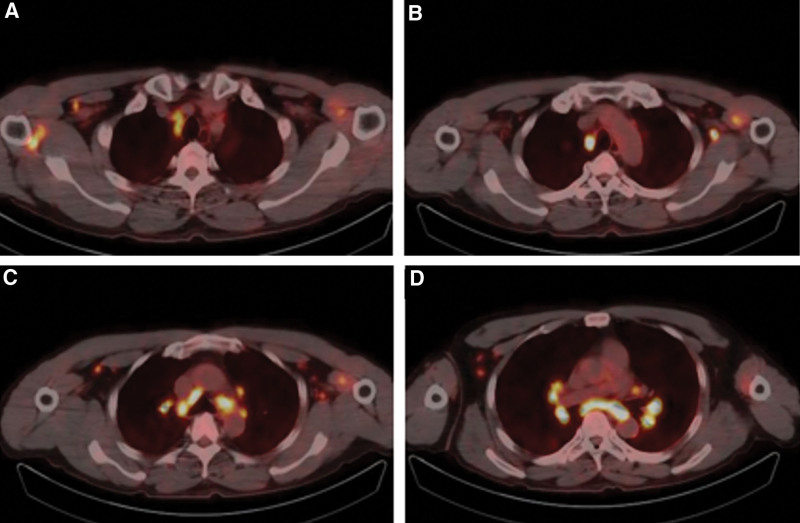
18F-FDG-PET imaging after five cycles demonstrating bilateral supraclavicular, mediastinal, bilateral hilar, bilateral axillary lymph nodes were enlarged with hypermetabolism, the right interlobar pleural nodules were slightly higher, the subpleural metabolism of both lungs was slightly higher. FDG = fluorodeoxyglucose, PET = positron emission tomography.

There were small patches of ground glass density scattered in both lungs, and nodules were seen in the right interlobar pleural area. Fibrous foci can be seen in the lower lobe of both lungs, and enlarged lymph nodes in the hepatogastric ligament in the abdominal cavity. The treatment was continued, and CT scan after 4 cycles showed the disease was stable. After the fifth cycle, the patient developed fever and diarrhea. Fluorodeoxyglucose-positron emission tomography scan showed the metastasis of lower thoracic esophageal cancer in left supraclavicular, abdominal and retroperitoneal lymph nodes was improved, and the lesion disappeared without hypermetabolism. At the same time, bilateral supraclavicular, mediastinal, bilateral hilar, bilateral axillary and celiac lymph nodes were enlarged with hypermetabolism, the right interlobar pleural nodules were slightly higher, the subpleural metabolism of both lungs was slightly higher, and the spleen was highly metabolized (Figs. 2, 4).

**Figure 5.**
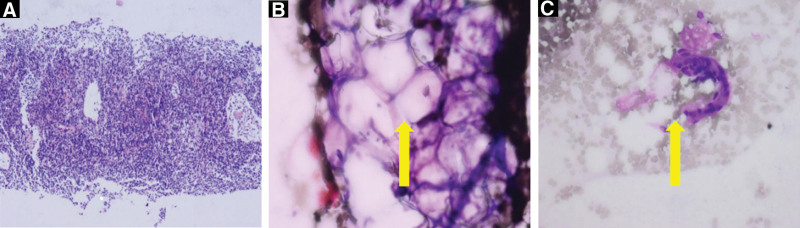
(A) Axillary lymph node biopsy showing normal lymphoid tissue. (B) (C) Wright-Giemsa staining showing blood and interstitial components (yellow arrows), no metastatic cancer was found.

Enlarged lymph node biopsy showed lymphoid tissue, and Wright-Giemsa staining showed blood and interstitial components, no metastatic cancer was found (Fig. 5).

**Figure 6.**
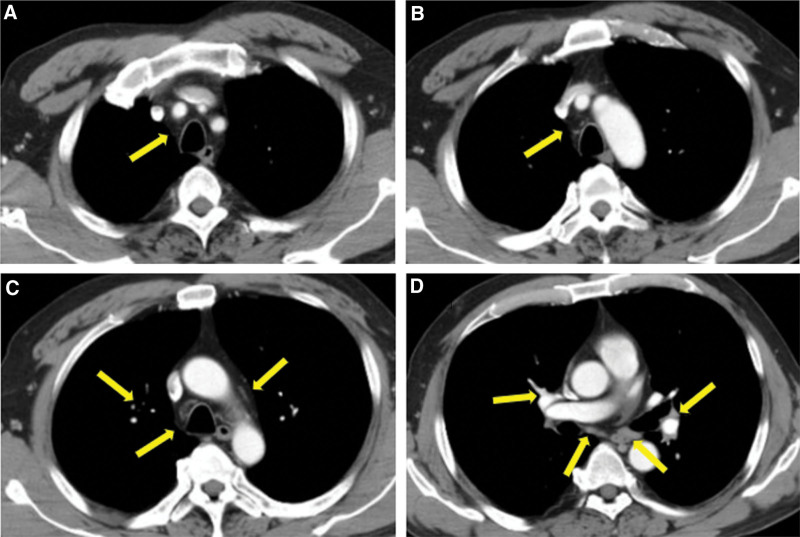
Computed tomography scan after two months of the administration of corticosteroid demonstrating the enlargement of lymph nodes shrinkage (yellow arrows).

Considering the medical history, a diagnosis of sarcoidosis-like reaction induced by immunotherapy was prescribed. Oral prednisolone was started at 80 mg/d, and the clinical course was rapidly favorable. CT scan after 2 months showed the enlargement of hilar and mediastinal lymph nodes shrinkage (Fig. 6).

The enlarged lymph nodes in the neck disappeared, no abnormality was found in the abdominal organs, and no enlarged lymph nodes were found in the retroperitoneal cavity.

Subsequently, the patient received 1 year of follow-up antitumor treatment due to the appearance of sarcoidosis-like reactions in the lungs. The patient had a good prognosis and is currently in a stable condition.

## 3. Discussion

Sintilimab has been used as a clinical therapeutic strategy of a variety of malignant tumors. According to the results of Shen et al, the overall median progression-free survival: 7.2 versus 5.7 months of unresectable locally advanced, recurrent or metastatic ESCC treated with sintilimab combined with chemotherapy (hazard ratio [HR] 0.558, 95% confidence interval [CI] 0.461–0.676, *P* < .0001). Hazard ratios (95% CI) were estimated with a stratified Cox proportional hazards model where the stratification factors were PD-L1 CPS, ECOG performance status score, chemotherapy regimen, and hepatic metastasis. For programmed death ligand 1 positive population: 8.3 versus 6.4 months (HR 0.580, 95% CI 0.449–0.749, *P* < .0001).^[7]^

IrAE usually involves multiple organs and systems, and has the most common impact on thoracic lymph nodes and lungs in reported cases, followed by skin. Can be characterized by sarcoidosis-like reactions, reactive lymphadenopathy, skin lesions (such as plaques and nodules), gastrointestinal manifestations (such as colitis) and endocrine disorders (including hypophysitis and hypothyroidism) (Table 1)

**Table 1:** Reported immune checkpoint inhibitor-induced sarcoidosis-like drug reaction (N = 29).

Age (Range, mean, median)	37–85, 57.8, 56
Gender (N, %)	Female (14, 48%), male (15, 52%)
Malignancy (N, %)	Melanoma (17, 59%), lung carcinoma (5,17%), breast carcinoma (2, 7%), lymphoma (1,3%), skin carcinoma (1, 3%), renal carcinoma (1, 3%), urothelial carcinoma (1, 3%), uterine leiomyosarcoma (1, 3%)
Drug (N, %)	Ipilimumab (7, 24%), nivolumab (5, 17%), pembrolizumab (5,17%), durvalumab (1,3%), cemiplimab (1,3%), atezolizumab (2,7%), Ipilimumab combined with nivolumab or pembrolizumab (8, 28%)
Weeks on therapy until drug reaction (range, median)	1–29, 8
Body parts involved (N, %)	Mediastinal and hilar lymph node (21, 72%), lung (9,31%), skin (7,24%), digestive tract (3,10%), other lymph nodes (2, 7%), eye (1, 3%), thyroid (1,3%), pleura (1,3%), spleen (1,3%), kidney (1,3%)
Treatment strategies (N, %)	Discontinuation of immunotherapy (9, 31%), corticosteroid therapy (11, 38%), Discontinuation of immunotherapy and corticosteroid therapy (2, 7%), lymph node dissection (1, 3%), no special treatment or unknown (6, 21%)
Outcome (N, %)	Complete resolution (4, 14%), improvement (15, 52%), stabilization (10, 34%)

The pathogenesis of sarcoidosis is very complex, and it is not clear whether there is a causal relationship between sarcoidosis-like reaction and primary tumor, or whether it interacts with primary tumor.

There are several theories about the pathogenesis of local sarcoidosis-like reaction in the lung: Anti-PD-1 antibodies produce objective reactions in about 20% to 25% of patients with non-small cell lung cancer, melanoma or renal cancer.^[8]^ However, the adverse events do not seem to hinder their curative effect. Therefore, we speculate that the sarcoidosis-like reaction may be the immune defense response against tumor cells. Das et al^[9]^ reviewed a large number of clinical ipilimumab studies and found that the emergence of irAEs usually indicates a better clinical response. In another retrospective study of 119 patients who received anti cytotoxic T-lymphocyte antigen 4 treatment, 20 patients with imaging manifestations of irAEs (including sarcoidosis-like reactions) had better responses than other 99 patients without irAEs: the disease control rate and complete remission rate in the irAE group were 55% and 25%, while those without irAEs were 10% and 3% respectively.^[10]^

In addition, June et al^[11]^ believe that sarcoidosis-like reaction is a T-cell-mediated immune response. Related studies have found that the sarcoidosis-like reaction after immunotherapy is mainly T-helper1 cell response mediated by interleukin-2 and IFN- γ, and the increase in the number and function of Th17 cells in tumor patients receiving anti-ICIs therapy.^[12]^ Th17 cells can promote the development of sarcoidosis fibrosis and play an indispensable role in sarcoidosis-like granuloma formation. Long-term antigen stimulation causes a series of T-lymphocyte immune response, which stimulates monocytes to form epithelioid granuloma.

Some studies suggest that the occurrence of sarcoidosis is related to age, environmental and genetic factors, and familial inheritance. In addition, some research supports that mycobacterium tuberculosis infection, acne, and even certain fungi may be the cause of sarcoidosis; certain occupations or exposure to toxins such as pesticides are also risk factors for sarcoidosis.^[13]^

There are both similarities and differences between ICI-induced sarcoidosis and typical sarcoidosis. The histopathology of the reported cases of sarcoidosis induced by ICI is exactly the same as that of sarcoidosis. Biopsy revealed focal infiltrative lesions of non-caseous epithelioid granuloma and giant cell granuloma, and these lesions can be integrated into micro-nodules. In addition, sarcoidosis-like reaction has the manifestation of organizing pneumonia which is different from sarcoidosis: cough, fever, bilateral diffuse shadow, ground glass shadow, nodular shadow, especially in the inferior field of the lung.

The staging and therapeutic strategies of sarcoidosis-like reactions depends on the severity of systemic symptoms and organ injury.^[14]^ About half of the patients with sarcoidosis-like reactions do not need treatment, and it is necessary to temporarily use corticosteroids for local or systemic treatment only when organ function is affected or when the patients have serious clinical symptoms. In reported cases, sarcoidosis-like reactions responded well to treatment (Table 1).

Most patients who develop sarcoid-like reactions after immunotherapy can be cured with timely treatment. However, reapplication of ICI may lead to recurrence of sarcoidosis, with different sites and severity than the initial presentation, but the treatment approach is usually similar. Therefore, clinicians need to carefully evaluate the benefits and risks to the patient before deciding whether to continue ICI therapy.

Immune therapy has profoundly changed the landscape of cancer treatment and brought new hope to cancer patients. However, as the use of ICI in cancer therapy increases, the incidence of irAEs and sarcoid-like reactions will also increase accordingly. From this perspective, it is an urgent problem to achieve maximum therapeutic efficacy while avoiding the occurrence of irAEs as much as possible. This requires clinical physicians to have a clear understanding of the mechanisms of related diseases and to be conscious of irAEs during diagnosis and treatment. Moreover, more extensive and in-depth research on related diseases and treatment strategies is needed in the future.

## Author contributions

**Conceptualization:** Linlin Wang.

**Methodology:** Haoqian Li, Fengchun Mu, Bing Zou.

**Project administration:** Haoqian Li.

**Visualization:** Fengchun Mu, Bing Zou, Linlin Wang.

**Writing – original draft:** Haoqian Li.

**Writing – review & editing:** Haoqian Li.
